# Does social intolerance vary according to cognitive styles, genetic cognitive capacity, or education?

**DOI:** 10.1002/brb3.2704

**Published:** 2022-09-01

**Authors:** Aino Saarinen, Liisa Keltikangas‐Järvinen, Henrik Dobewall, C. Robert Cloninger, Ari Ahola‐Olli, Terho Lehtimäki, Nina Hutri‐Kähönen, Olli Raitakari, Suvi Rovio, Niklas Ravaja

**Affiliations:** ^1^ Faculty of Medicine Department of Psychology and Logopedics University of Helsinki Helsinki Finland; ^2^ Research Unit of Psychology University of Oulu Oulu Finland; ^3^ Department of Clinical Chemistry Fimlab Laboratories and Finnish Cardiovascular Research Center Tampere Finland; ^4^ Department of Psychiatry Washington University St. Louis Missouri; ^5^ Department of Internal Medicine Satasairaala Central Hospital Pori Finland; ^6^ Psychiatric and Neurodevelopmental Genetics Unit, Department of Psychiatry Massachusetts General Hospital Boston Massachusetts; ^7^ Institute for Molecular Medicine Finland (FIMM) University of Helsinki Helsinki Finland; ^8^ Faculty of Medicine and Health Technology Tampere University Tampere Finland; ^9^ Tampere Centre for Skills Training and Simulation Tampere University Tampere Finland; ^10^ Centre for Population Health Research University of Turku and Turku University Hospital Turku Finland; ^11^ Research Centre of Applied and Preventive Cardiovascular Medicine University of Turku Turku Finland; ^12^ Department of Clinical Physiology and Nuclear Medicine Turku University Hospital Turku Finland

**Keywords:** cognition, cognitive performance, intelligence, longitudinal, prejudice

## Abstract

**Background:**

Low education, low cognitive abilities, and certain cognitive styles are suggested to predispose to social intolerance and prejudices. Evidence is, however, restricted by comparatively small samples, neglect of confounding variables and genetic factors, and a narrow focus on a single sort of prejudice. We investigated the relationships of education, polygenic cognitive potential, cognitive performance, and cognitive styles with social intolerance in adulthood over a 15‐year follow‐up.

**Methods:**

We used data from the prospective population‐based Young Finns Study (*n* = 960‒1679). Social intolerance was evaluated with the Social Intolerance Scale in 1997, 2001, and 2011; cognitive performance with the Cambridge Neuropsychological Test Automated Battery in 2011; cognitive styles in 1997; and socioeconomic factors in 1980 (childhood) and 2011 (adulthood); and polygenic cognitive potential was calculated based on genome‐wide association studies.

**Results:**

We found that nonrational thinking, polygenic cognitive potential, cognitive performance, or socioeconomic factors were not related to social intolerance. Regarding cognitive styles, low flexibility (*B* = –0.759, *p* < .001), high perseverance (*B* = 1.245, *p* < .001), and low persistence (*B* = –0.329, *p* < .001) predicted higher social intolerance consistently in the analyses.

**Discussion:**

When developing prejudice‐reduction interventions, it should be considered that educational level or cognitive performance may not be crucial for development of social intolerance. Adopting certain cognitive styles may play more important roles in development of social intolerance.

## 1 INTRODUCTION

Social intolerance refers to intolerance toward others’ different attitudes, lifestyles, cultures, or values. It predisposes to prejudices that, in turn, are defined as negative evaluations or affective responses toward outgroup members (e.g., individuals with different political, religious, or sexual orientation, or ethnic background) (Amodio, 2014). Prejudices are related to a variety of adverse outcomes such as discriminative behaviors (Talaska et al., 2008), less favorable treatment decisions about outgroup members’ health conditions (Kaseweter et al., 2012), and higher readiness to assign criminal punishments to outgroup members (Johnson et al., 2012). Although many prejudice‐reduction interventions have been developed, some interventions may, in fact, even increase prejudices among subjects with prejudice‐prone ideological attitudes (e.g., social dominance) (Asbrock et al., 2012) or high baseline level of prejudices (Vorauer & Sasaki, 2010). Hence, a deeper understanding of the roots of social intolerance could provide novel possibilities for tailoring more effective interventions.

To date, a variety of psychological factors has been proposed to predispose to social intolerance, including parents’ ideologies in childhood, threat perceptions, anxiety proneness, aspects of moral development, religious beliefs, and personality traits such as openness to experience and agreeableness (Dhont & Hodson, 2014; Meeusen & Dhont, 2015; Rowatt et al., 2014; Sibley & Duckitt, 2008). Single risk factors have been integrated in a dual‐process motivational (DPM) model proposing that personality dimensions and social environment (e.g., inequality) play roles in the development of ideological attitudes such as right‐wing authoritarianism (i.e., values of conformity, traditionalism) and social dominance (i.e., a desire to see one's in‐group dominating one's outgroup) (Duckitt & Sibley, 2010). Those ideological attitudes, in turn, increase susceptibility to perceptions of threat and competition between groups and, eventually, predispose to prejudices (Duckitt & Sibley, 2010). The model has gained also empirical support (Duckitt & Sibley, 2007; McFarland, 2010). The aim of the current study was to focus on three sorts of plausible risk factors for developing prejudices: (1) socioeconomic position (i.e., educational level, level of income), (2) cognitive styles (i.e., how an individual prefers to process acquired knowledge), and (3) cognitive performance (i.e., one's capacity for information processing).<COMP: Please set reference citations as per the journal style, that is, in alphabetical order.>

Regarding educational level, education is postulated to increase knowledge about different groups of people with different values and lifestyles, to alleviate fear of uncertainty, and to promote openness to new experiences (Vogt, 1997), thus lowering likelihood for social intolerance. Current research literature suggests that a low educational level may increase susceptibility to higher social intolerance. Two studies of a longitudinal data set have found that low educational level predicts lower levels of liberal and antiracist political attitudes in adulthood (Deary et al., 2008; Schoon et al., 2010). Additionally, prior cross‐sectional findings suggest that low educational level is related to higher need for ethnic distance (i.e., a lower intention to avoid social contacts with ethnic minorities) (Hello et al., 2006) and higher ethnocentrism (Meeusen et al., 2013). Overall, an array of educational programs has been launched for both children and adults, with an aim to reduce social intolerance by increasing knowledge about characteristics of various cultures, values, and minorities (Rutland & Killen, 2015). All these educational programs have been based on the assumption that increasing knowledge about various minorities would reduce prejudices toward them.

While low educational level seems to relate to higher level of prejudices, an even stronger correlate of prejudices may be low cognitive abilities. Findings of a longitudinal data set have indicated that lower cognitive performance in childhood (at age of 10‒11 years) predicts lower social liberalism and lower antiracism in adulthood (at the ages of 30 and 33 years), partly via educational qualifications in adulthood (Deary et al., 2008; Hodson & Busseri, 2012; Schoon et al., 2010). Likewise, another longitudinal study has shown that lower intelligence in adolescence and adulthood predicts slightly less liberal values later in adulthood (Kanazawa, 2010). In addition, evidence from cross‐sectional studies suggests that lower cognitive abilities are related to higher need for ethnic distance (Hello et al., 2006), higher ethnocentrism (Meeusen et al., 2013), higher blatant and subtle prejudice (De Keersmaecker et al., 2018), and higher prejudice toward sexual minorities (Hodson & Busseri, 2012). Also, poor working memory is linked to progroup attitudes and violence endorsement against outgroups (Zmigrod et al., 2021). Low cognitive abilities may predispose to stronger social intolerance by restricting one's capacity to, for example, process complex information, to avoid simple categorization between social groups, or to regulate one's intuitive (not knowledge‐based) predispositions during decision making.

Regarding cognitive styles, it was stated already in the 1950s that “a person's prejudice is unlikely to be merely a specific attitude toward a specific group; it is more likely to be a reflection of his whole habit of thinking about the world” (Allport, 1954). Later on, in the 2010s, the Cognitive Ability and Style to Evaluation (CASE) model about prejudices was developed, postulating that certain cognitive styles may increase susceptibility to social intolerance and prejudice (Dhont & Hodson, 2014). To date, regarding cognitive styles and prejudices, most research has been directed to need for disclosure, rigidity, and spiritual thinking. First, “need for closure” refers to desire for order and predictability, a tendency to make conclusions impulsively, a discomfort with ambiguity, and closed‐mindedness (Roets & Van Hiel, 2007). Evidence from cross‐sectional studies suggests that high need for closure is at least indirectly associated with higher racism (Cornelis & Van Hiel, 2006; Roets & Van Hiel, 2006; Van Hiel et al., 2004) and higher blatant and subtle prejudice (De Keersmaecker et al., 2018). Also, impulsivity seems to play a role in extreme progroup attitudes (Zmigrod et al., 2021). Second, single findings suggest that high religiosity or spirituality may be associated with more negative attitudes toward outgroup members (Johnson et al., 2012) and that religious priming may increase prejudice (Yilmaz et al., 2016). Third, regarding rigidity, it has been found that high rigidity of thinking correlates with higher implicit racism (Cunningham et al., 2004), whereas high psychological flexibility is related to lower generalized prejudice (Levin et al., 2016). A review and original studies indicate that cognitive rigidity (or inflexibility) is associated with ideological extremism and dogmatism in political extreme ideologies (Zmigrod, 2020; Zmigrod et al., 2020), intellectual humility (Zmigrod et al., 2019), authoritarianism and nationalism (Zmigrod et al., 2018), and right‐wing attitudes (Van Hiel et al., 2016).

Taken together, there is evidence that low educational level, low cognitive abilities, and certain cognitive styles may act as susceptibility factors for development of social intolerance and prejudices. There are, however, a variety of severe limitations in previous research literature. These together lead to scientific gaps. First, many studies have included comparatively small or biased samples (e.g., college students), whereas population‐based samples have been largely lacking (Dhont & Hodson, 2014). Second, many studies have focused only a single factor without controlling for the other factors. For example, a review emphasized that “no single study has simultaneously investigated cognitive ability and style” (Hodson & Dhont, 2015). Noticeably, it has been noted that the association of cognitive abilities with social intolerance has commonly been investigated without controlling for educational level (Dhont & Hodson, 2014). Finally, several previous studies have investigated social intolerance or prejudices by defining a certain political orientation as a marker of prejudice (e.g., conservative values referring to higher social intolerance). There is evidence, however, that liberals and conservatives may have similar levels of intolerance toward ideologies that are contradictory to their own ideology (Brandt & Crawford, 2016; Brandt et al., 2014). Further, a review concluded that similar psychological characteristics (such as social dominance) seem to play roles in both left‐ and right‐wing political extremes (Zmigrod, 2020). Hence, a conceptually broader viewpoint to social intolerance (i.e., investigating social intolerance at a more general level without defining specific political values per se as prejudices) would provide a deeper understanding of the psychological mechanisms behind social intolerance.

To date, evidence about the role of genetic factors in social intolerance is very limited. To the best of our knowledge, only three studies have combined genetic and prejudice‐related factors. First, a twin study found that genetic factors explain approximately 32% of the variance in negative attitudes toward strangers (Kandler et al., 2015). Another twin study showed that social dominance orientation (i.e., a personality disposition strongly related to prejudices) is moderately heritable, with heritability estimates around 24%−37%, varying between subscales (Kleppesto et al., 2019). Finally, a candidate‐gene study found that certain environmental factors (e.g., negative contacts with outgroup members) predict intergroup bias more strongly in individuals with a certain variant of a serotonin transporter gene (5‐HTTLPR) (Cheon et al., 2014). While social science research usually tries to account for known environmental confounders, genetic risk factors are largely ignored (Harden & Koellinger, 2020; Mills & Tropf, 2020). This practice is likely to change over the next decade since there is consensus that nature and nurture interact in human development (Spinath et al., 2008; Zwir et al., 2019) and the increasing availability of reliable measures of genetic predisposition that explain a reasonably large part of the variance produced by the use of GWAS data involving hundreds of thousands individuals (Karlsson Linnér et al., 2019; Lee et al., 2018). The obtained polygenetic scores, however, can be applied in smaller, genetically informed samples to overcome this gap in the literature.

The aim of this study was to investigate the relationship of educational level, cognitive test performance, polygenic cognitive potential, and cognitive styles with social intolerance in adulthood. We leveraged the data from prospective population‐based Young Finns Study that provided exceptional possibilities to examine the trajectory of social intolerance over a 15‐year follow‐up. Cognitive performance was evaluated using the Cambridge Neuropsychological Test Automated Battery (CANTAB) including four subtests (Paired Associates Learning Test, Reaction Time Test, Rapid Visual Information Processing Test, and Spatial Working Memory Test). Cognitive styles included flexibility (ability to adapt one's behavior to unexpected changes of the situation or circumstances), perseverance (disposition to repeat a behavior despite changing circumstances), persistence (disposition to continue working toward the goals despite temporary frustration), distractibility (disposition to become interrupted by irrelevant stimuli), and nonrational thinking (beliefs in, e.g., sixth sense, extrasensory perception, telepath, or miracles).

## 2 MATERIAL AND METHODS

### 2.1 Participants

We used data from the prospective Young Finns Study. The participants were selected randomly from six age cohorts (born between 1962 and 1977) who were living in the surrounding regions of the Finnish universities with medical schools (Helsinki, Turku, Tampere, Kuopio, Oulu). The participants were selected using the population register of the Social Insurance Institution that covers the whole population of Finland. The original sample included 3596 participants (ethnic Finns) in the baseline measurement in 1980 (when participants were aged 3‒18 years). The participants have been followed since then so that the latest follow‐up measurement was in 2012 (participants were aged 35‒50 years). The study was carried out in accordance with the Declaration of Helsinki. The design of the Young Finns Study has been approved by all the Finnish universities with medical schools (i.e., University of Helsinki, University of Turku, University of Kuopio, University of Oulu, and University of Tampere) at the beginning of the study (in 1980), and the ethical permissions have been updated at the time of each follow‐up measurement. Before participation, all the participants or their parents (for participants aged below 12 years) provided informed consent after the nature of the study procedures had been fully explained. The design of the Young Finns Study is described with more detail elsewhere (Raitakari et al., 2008).

For this study, social intolerance was evaluated in 1997, 2001, and 2012; self‐reported cognitive style in 1997; cognitive performance in 2011; parents’ socioeconomic factors in 1980; and participants’ socioeconomic factors in 2011. The measurement years are summarized in Table 1. We included all the participants with data available on the study variables (in at least one measurement point as listed at the beginning of this paragraph) that were under investigation in each analysis. For example, participants who had not responded to the questionnaire of social intolerance in 1997, 2001, or 2012 were excluded from the analyses; or participants who had not performed cognitive tests in 2012 were excluded from those analyses. The final sample sizes ranged between 960 and 1679 in the analyses. The participants (57.9 % female, all of them were White by ethnicity) were on average 27.6 years old. The educational level of the participants was most typically high school or occupational school (61.2 %) or academic level (38.8 %).

**TABLE 1:** Study timeline: measurement years of the study variables

Study variable	1980	1997	2001	2007	2011/2012
Parent's socioeconomic position	X				
Participants’ socioeconomic position					X
Cognitive styles					
Flexibility		X			
Distractibility		X			
Perseverance		X			
Persistence		X			
Nonrational thinking		X			
Collection of genetic samples					X
Cognitive performance					X
Social intolerance		X	X		X

### 2.2 Measures

#### 2.2.1 Social intolerance

Social intolerance was evaluated with the Social Intolerance Scale of the Temperament and Character Inventory (TCI) (Cloninger et al., 1994). It includes 8 items (e.g., “Usually I can accept other people as such as they are, although they were very different from me” [reversed]; “People who do not accept my opinions make me angry” “Usually it is easy for me to like other people whose values are very different than mine” [reversed]) that are responded with a 5‐point scale (1 = totally disagree; 5 = totally agree). The internal reliability of the scale was good (Cronbach's *α *= .77‒.78 in 1997, 2001, and 2012). We calculated the mean score of the items for each measurement year (1997, 2001, and 2012) for all the participants who had responded to at least 50% of the items. Finally, the scores for social intolerance were standardized with the mean and SD of the first measurement year (1997), in order to have a stable scale for the variables between different measurement years. The internal consistency of the scale has been found to be adequate also previously (Fossati et al., 2007; Jylhä & Isometsä, 2006; Snopek et al., 2012).

#### 2.2.2 Cognitive styles

Cognitive styles included flexibility, distractibility, persistence, perseverance, and nonrational thinking.

Distractibility, flexibility, and persistence were evaluated with the Revised Dimensions of Temperament Survey (Windle & Lerner, 1986). The scale of *distractibility* includes four items that measure the disposition to become interrupted by irrelevant internal and external stimuli and to easily direct attention away from the task along with other stimuli (e.g., “When I'm concentrating on a task, any environmental stimuli cannot catch my attention”; “When I am doing something, other things can easily get me to direct my attention elsewhere” [reversed]). The scale of *flexibility* includes six items that assess the ability to adapt one's behavior to unexpected changes of the situation or circumstances (e.g., “Changes in my plans make me nervous”; “I resist changes in my daily program”). The scale of *persistence* consists of three items that measure the disposition to continue working toward the goals despite temporary frustration or challenges (e.g., “I usually continue working until I have completed the task”; “I can continue working with the same task for a long time”). All the items were responded with a 5‐point scale (1 = totally disagree; 5 = totally agree). In this study, the internal consistencies of the scales were adequate for the scales of distractibility (*α = *.79), perseverance, (*α = *.72), and flexibility (*α = *.69). Internal consistency of persistence was lower (*α = *.56) that may result from the low number of items. The correlations between single items and the mean score were high (all the correlations were *r *= 0.72–0.74). Furthermore, the stability of the scales is shown to be adequate (Windle & Windle, 2006).

*Perseverance* was measured with the Formal Characteristics of Behavior—Temperament Inventory (Strelau & Zawadzki, 1993). The scale of perseverance measures the disposition to repeat a behavior despite changing circumstances (even when that behavior would not be situationally appropriate), or the inability to direct attention to novel targets in line with the circumstances (e.g., to rethink previous decisions again or get stuck into a working phase). The scale consists of 20 items (e.g., “After completing a time‐taking task, I shortly stop thinking about it”; “Usually I do not start rethinking about the decisions that I have made previously” [reversed]; or “It is common that a certain issue is bothering me”) that were responded with no (score 0) or yes (score 1). The internal consistency of the scale was adequate (Cronbach's *α = *.70). Furthermore, previous studies have confirmed the validity, stability, and internal reliability of the scale (De Pascalis et al., 2000; Strelau & Zawadzki, 1993; Strelau & Zawadzki, 1995).

*Mystical thinking* was evaluated with the subscale of Spiritual Acceptance (vs. Rational Materialism) of the Temperament and Character Inventory (TCI) (Cloninger et al., 1994). The scale includes 13 items (e.g., “I believe that miracles can happen”; “Sometimes I know what will happen because I have a ‘sixth sense’”; “I believe that extrasensory perception [e.g., telepathy or forecasting] really are possible”; “Commonly I am interested about things that cannot be scientifically explained”) that are rated with a 5‐point scale (1 = totally disagree; 5 = totally agree). The internal reliability of the scale was very good (*α = *.89).

We calculated the mean scores of flexibility, persistence, distractibility, perseverance, and nonrational thinking for all the participants who had responded to at least 50% of the items.

#### 2.2.3 Cognitive performance

Cognitive performance was measured in 2011 with four subtests of the Cambridge Neuropsychological Test Automated Battery (CANTAB): (1) Paired Associates Learning Test (i.e., visual episodic memory and visuospatial associative learning), (2) Reaction Time Test (i.e., reaction time and response accuracy), (3) Rapid Visual Information Processing Test (i.e., sustained visual attention), and (4) Spatial Working Memory Test (i.e., spatial working memory). The tests include a computerized set of items (approximately 20–30 min).

From each cognitive subtest, several variables were collected (e.g., number of correct and incorrect responses). Each single variable was classified into four classes (1–4) on the basis of quartiles (normally distributed variables) or quartile‐like groups (nonnormally distributed variables). Next, all the single variables within each subtest were summed together to form scores of each cognitive subtest (only such variables were included that discriminated between participants). Finally, the sum scores were rank‐order normalized (mean = 0, SD = 1). The sum scores were included as continuous variables in the analyses. A more detailed description of the procedure of the cognitive tests is available elsewhere (Rovio et al., 2016). Taken together, for each participant, we formed altogether four scores for cognitive performance: a score for the Paired Associates Learning Test, a score for the Reaction Time Test, a score for the Rapid Visual Information Processing Test, and a score for the Spatial Working Memory Test.

#### 2.2.4 Socioeconomic factors

*Socioeconomic factors* included participants’ and their parents’ level of income and educational level. Participants’ and their parents’ educational level was categorized into three categories (1 = comprehensive school; 2 = high school or occupational school; 3 = academic level, that is, university or college). If mother's and father's educational levels differed from each other, we selected the higher level of education. Level of parents’ income (in 1980) included eight categories (1 = less than 15,000 Finnish mark per year; 8 = more than 100,000 Finnish mark per year). Participants’ level of income (in 2011) was evaluated with a 13‐point scale (1 = less than 5000€ per year; 13 = more than 60,000€ per year).

#### 2.2.5 Polygenic cognitive potential

A polygenic score for cognitive performance was calculated for each participant. The genotyping was performed for 2443 samples using a custom build Illumina Human 670k BeadChip at Welcome Trust Sanger Institute. Genotypes were called using Illuminus clustering algorithm (Teo et al., 2007). Genotype imputation was conducted using Beagle software (Browning et al., 2018) and The Sequencing Initiative Suomi (SISu) as the reference data. A polygenic score for the cognitive function was calculated using LDpred, a Bayesian method that estimates posterior mean causal effect sizes from genome‐wide association (GWA) study summary statistics by assuming a prior for the genetic architecture and linkage disequilibrium (LD) information from a reference panel (Vilhjalmsson et al., 2015): an infinitesimal fraction of causal variants was assumed, and summary statistics from Savage et al.’s (2018) GWA study for intelligence were used. The LD between markers was estimated from the SISu data.

### 2.3 Statistical analyses

Statistical analyses were conducted with STATA SE (version 13.0). First, we compared included (*n* = 1764) and excluded participants (*n* = 1832) using independent samples *t*‐tests and chi‐square tests.

Second, we examined the association of socioeconomic factors with social intolerance. The dependent variable was social intolerance in 2012 and the independent variables included level of income in childhood and parents’ educational level in 1980 (Model 1) and also participants’ level of income and educational level in adulthood in 2012 (Model 2). The models were adjusted for age and sex. There was not any significant multicollinearity in the models (VIF values between 1.23 and 1.24).

Third, we examined the associations of polygenic cognitive potential (genetic samples collected in 2011) and cognitive test performance (assessed in 2011) with social intolerance using linear regression analysis. The dependent variable was social intolerance in 2012. The independent variables included polygenic cognitive potential (Model 1) and also performance in different cognitive domains including (i) visual memory and visuospatial associative learning, (ii) reaction time, (iii) sustained visual attention, and (iv) spatial working memory (Model 2). In both models, covariates included age, sex, and participants’ and their parents’ socioeconomic factors. There was no significant multicollinearity between various cognitive tests (as evaluated with VIF values).

Finally, the associations of cognitive styles with social intolerance were investigated using growth curve models. Growth curve models estimate (1) “fixed effects” that are interpreted as classic regression coefficients and (2) “random effects” that estimate individual‐level variance in the intercept, slopes, and residual variance (i.e., within‐individual variance over the follow‐up time). We predicted the trajectory of social intolerance over the 15‐year follow‐up time (in 1997, 2001, and 2012) by cognitive styles. All the models were adjusted for follow‐up time, follow‐up time squared, age, sex, and participants’ and their parents’ socioeconomic factors. In addition, all the models included the interactions of follow‐up time with cognitive styles, in order to investigate whether the associations of cognitive styles with social intolerance change over the follow‐up. Each cognitive style (flexibility, persistence, distractibility, perseverance, and nonrational thinking) was set as predictor separately. This was done to avoid excessive number of independent variables and interactions in the model (i.e., besides of the main effects, there were interactions of predictor with follow‐up time and follow‐up time squared).

## 3 RESULTS

### 3.1 Descriptive information

Descriptive statistics of the study variables are presented in Table 2. The results of attrition analyses are described in Supplementary Material. Educational level and measures of cognitive performance were positively correlated in the following way: education and the Rapid Visual Information Processing Test (*r* = 0.26), education and the Spatial Working Memory Test (*r* = 0.10), education and the Paired Associates Learning Test (*r* = 0.15), and education and the Reaction Time Test (*r* = 0.09).

**TABLE 2:** The means, standard deviations (SD), frequencies, and ranges of the study variables

	Mean	*SD*	Frequency (%)
Age (1997)	27.60	5.00	
Sex (female)			1107 (57.9)
Parents’ educational level (1980)			
Comprehensive school			629 (32.9)
High school or occupational school			791 (41.4)
Academic level			491 (25.7)
Family income in childhood (1980)	4.88	1.94	
Participants’ educational level (2011)			
Comprehensive school			144 (8.3)
High school or occupational school			918 (61.2)
Academic level			674 (38.8)
Participants’ level of income (2011)	7.34	3.08	
Cognitive styles (1997)			
Flexibility	3.92	0.61	
Distractibility	3.02	0.73	
Perseverance	0.58	0.19	
Persistence	3.69	0.66	
Nonrational thinking	2.69	0.80	
Polygenic cognitive potential	0.34	1.01	
Cognitive performance (CANTAB) (2011)			
Paired Associates Learning Test	0.03	0.98	
Spatial Working Memory Test	0.01	0.99	
Rapid Visual Information Processing Test	0.05	1.00	
Reaction Time Test	0.01	0.99	
Social intolerance			
1997	2.14	0.52	
2001	2.12	0.52	
2011	2.17	0.49	

### 3.2 Socioeconomic factors and social intolerance

Table 3 shows the findings of regression analyses when predicting social intolerance by socioeconomic factors both in childhood and adulthood. Level of family income in childhood or participants’ or their parents’ educational level were not related to social intolerance. Participants’ high level of income in adulthood was related to slightly lower social intolerance (*p* < .014) but this did not survive after Bonferroni correction for multiple testing.

**TABLE 3:** Results of regression analyses when predicting social intolerance by socioeconomic factors

	Model 1 (*n* = 1679)			Model 2 (*n* = 1249)		
	*B*	95% CI	*p*	*B*	95% CI	*p*
Family income in childhood	−0.004	−0.018; 0.009	.565	0.000	−0.017; 0.016	.956
Parents’ educational level	−0.015	−0.051; 0.021	.420	0.014	−0.029; 0.057	.517
Participants’ level of income				−0.012	−0.022; −0.003	.014
Participants’ educational level				−0.037	−0.093; 0.019	.194

*Note*: Adjusted for participants’ age and sex.

### 3.3 Polygenic cognitive potential, cognitive test performance, and social intolerance

The results of regression analyses examining the associations of cognitive test performance and polygenic cognitive potential with social intolerance are presented in Table 4. No significant associations were found. That is, neither polygenic cognitive potential nor performance in any cognitive domain (in visual episodic memory and visuospatial associative learning, or reaction time, or sustained visual attention, or spatial working memory) was related to social intolerance. As additional analyses, we reran the analysis without controlling for socioeconomic factors, but all the associations of cognitive performance in different subtests with social intolerance remained nonsignificant.

**TABLE 4:** Results of regression analyses when predicting social intolerance by cognitive performance

	Model 1 (*n* = 1099)			Model 2 (*n* = 960)		
	*B*	95% CI	*p*	*B*	95% CI	*p*
Polygenic cognitive potential	0.028	−0.001; 0.057	.060	.025	−0.007; 0.057	.125
Paired Associates Learning Test				−.002	−0.037; 0.033	.911
Spatial Working Memory Test				−.005	−0.039; 0.029	.761
Rapid Visual Information Processing Test				.018	−0.016; 0.052	.308
Reaction Time Test				−.020	−0.052; 0.011	.204

*Note*: Adjusted for age, sex, and socioeconomic factors in childhood and adulthood.

### 3.4 Cognitive styles and social intolerance

**FIGURE 1:**
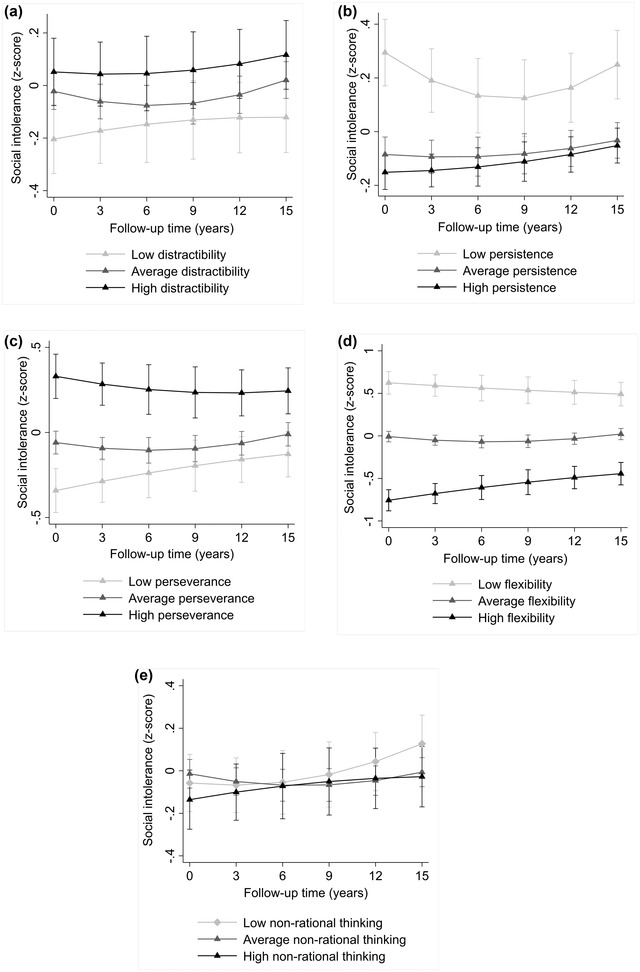
The trajectories of social intolerance in adulthood separately for participants with low (−1 SD), average, and high (+1 SD) scores of (a) distractibility, (b) persistence, (c) perseverance, (d) flexibility, and (e) nonrational thinking. Estimated means with 95% confidence intervals. *Note*: adjusted for age, sex, and participants’ and their parents’ socioeconomic factors

The results of growth curve models are presented in Table 5. The findings are illustrated in Figure 1. High distractibility, low persistence, high perseverance, and low flexibility predicted higher trajectory of social intolerance. Nonrational thinking, in turn, was not related to the trajectory of social intolerance. Figure 1 suggested that there may be a “threshold effect” in the associations of persistence and perseverance with social intolerance: participants with high persistence seemed to differ from participants with low persistence but not from participants with average levels of persistence. Similarly, participants with low perseverance seemed to differ from participants with high perseverance but not from participants with average levels of perseverance. In addition, there were no significant interactions between the predictors and follow‐up time or follow‐up time squared. This indicated that the associations of high distractibility, high perseverance, and low flexibility with higher social intolerance were evident over the 15‐year follow‐up.

**TABLE 5:** Results of the growth curve models. unstandardized estimates (*B*) with *p* values (within parentheses) of distractibility, persistence, perseverance, flexibility, nonrational thinking, and follow‐up time, when predicting the growth curve of social intolerance in adulthood

	Fixed effects					Random effects		
Predictor under investigation	Main effect of predictor (*p*)	Main effect of time (*p*)	Main effect of time^2^ (*p*)	Interaction between predictor and time (*p*)	Interaction between predictor and time^2^ (*p*)	Variance of intercept (*p*)	Variance of time (*p*)	Residual variance (*p*)
Distractibility	0.110 (.005)	−0.002 (.957)	0.000 (.848)	−0.003 (.818)	0.000 (.823)	0.803 (<.05)	0.029 (<.05)	0.559 (<.05)
Persistence	−0.329 (<.001)	−0.095 (.041)	0.005 (.116)	0.023 (.063)	−0.001 (.196)	0.781 (<.05)	0.029 (<.05)	0.558 (<.05)
Perseverance	1.245 (<.001)	0.028 (.284)	−0.005 (.758)	−0.065 (.134)	0.002 (.380)	0.777 (<.05)	0.028 (<.05)	0.559 (<.05)
Flexibility	−0.759 (<.001)	−0.137 (.009)	0.005 (.102)	0.033 (.013)	−0.001 (.165)	0.675 (<.05)	0.027 (<.05)	0.558 (<.05)
Nonrational thinking	−0.001 (.984)	−0.021 (.447)	0.002 (.233)	0.004 (.657)	0.000 (.466)	0.807 (<.05)	0.029 (<.05)	0.559 (<.05)

*n* = 1209 adjusted for age, sex, and participants’ and their parents’ socioeconomic factors.

*Note*: STATA does not report the exact *p* values for random effects.

As additional analyses, we examined whether educational level could modify the effects of cognitive styles on social intolerance. That is, we added the interaction between each cognitive style and education to the predictors. There were no significant interactions, indicating that the effect of each cognitive style on social intolerance was similar at different educational levels.

### 3.5 All predictors included simultaneously when predicting the trajectory of social intolerance

As additional analysis, we included all the predictor variables in a same growth curve model when predicting the trajectory of social intolerance. That is, the predictors included cognitive styles (distractibility, persistence, perseverance, flexibility, nonrational thinking), cognitive performance (visual episodic memory and visuospatial associative learning, or reaction time, or sustained visual attention, or spatial working memory), socioeconomic factors in childhood and adulthood (level of income, educational level), and polygenic cognitive potential. The analyses were adjusted for age and sex. The results are presented in Supplementary Table S1. To summarize, the only significant predictors of higher trajectory of social intolerance were low persistence, low flexibility, and high perseverance. Thus, the results remained similar to the main analyses, except for distractibility that became a nonsignificant predictor.

## 4 DISCUSSION

In this study, we investigated factors that could contribute to the trajectory of social intolerance over a 15‐year follow‐up in a population‐based Finnish sample. In summary, the results showed that cognitive styles were found to be the only predictors of social intolerance: low persistence, high perseverance, and low flexibility predicted higher trajectory of social intolerance consistently in the analyses. Spiritual (vs. rational) thinking did not predict social intolerance. Neither cognitive performance nor polygenic cognitive potential was related to social intolerance. Further, participants’ or their parents’ educational level was not significantly associated with participants’ social intolerance. Taken together, this study suggests that educational level or cognitive performance may not be crucial contributors for the development of social intolerance. Instead, certain cognitive styles may strongly predict higher trajectory of social intolerance in adulthood.

There are findings suggesting that high cognitive abilities are related to lower prejudices, lower ethnic distance, lower ethnocentrism, higher liberal values, and violence endorsement against outgroups (Meeusen & Dhont, 2015; Deary et al., 2008; Schoon et al., 2010; Hodson & Busseri, 2012; De Keersmaecker et al., 2018; Zmigrod et al., 2021). In our study, however, neither cognitive performance nor polygenic cognitive potential was associated with social intolerance. This may have several explanations. First, although some studies have obtained statistically significant associations between cognitive performance and prejudices, cognitive abilities may explain only a small portion of variation in prejudices: for example, circa 3% of prejudices toward sexual minorities (Keiller, 2010). Second, cognitive abilities may relate to better abilities to hide socially undesirable attitudes such as prejudices (Deary et al., 2008). Specifically, high cognitive abilities associate with lower explicit prejudice but higher implicit prejudices toward foreigners (von Stülpnagel & Steffens, 2010). In line with this, high cognitive abilities are related to lower racial prejudices and stronger support for racial equality *in principle* but not *in practice* (Wodtke, 2016).

Regarding cognitive styles, it has been found that high psychological rigidity or low flexibility correlates with lower implicit racism (Cunningham et al., 2004), higher generalized prejudice (Levin et al., 2016), dogmatism in political extreme ideologies (Zmigrod, 2020; Zmigrod et al., 2020), intellectual humility (Zmigrod et al., 2019), authoritarianism and nationalism (Zmigrod et al., 2018), and right‐wing attitudes (Van Hiel et al., 2016). Our population‐based study provided longitudinal evidence showing that high perseverance, low psychological flexibility, and low persistence predicted higher trajectory of social intolerance over a 15‐year follow‐up. Taken together, this suggests that there is an elevated likelihood for social intolerance in individuals with strong dispositions to (i) stop working when facing temporary frustration or challenges (e.g., proneness to make impulsive conclusions if experiencing frustration in interaction with outgroup members or if information processing is experienced as complicated), (ii) to refuse to revise one's beliefs rather than to flexibly rethink one's attitudes (e.g., one's beliefs about outgroup members may not be updated if encountering new pieces of information that are contradictory to one's previous observations), and (iii) not to adapt one's behavior to unexpected changes of circumstances (e.g., one's beliefs remain similar even if outgroup members change their behavior). Our findings provide empirical support for a framework of prejudices proposing that cognitive styles may lead to a biased assessment (threat perceptions), biased immediate responses (e.g., approach vs. avoidance when encountering outgroup members), and in that way to increase prejudices (Dhont & Hodson, 2014).

Single findings suggest that high religiosity or spirituality may be associated with prejudice and outgroup derogation (Johnson et al., 2012; Yilmaz et al., 2016). We found, however, no link between nonrational (vs. spiritual) thinking and social intolerance. It is necessary to note that some previous studies have examined prejudices toward such behaviors or values that are a part of individual's religious conviction: for example, intolerance toward atheists or gay men among Christians (Johnson et al., 2012). Instead, we examined social intolerance at a general level, that is, without defining a certain outgroup and without conceptual overlap between spiritual thinking and social intolerance. Moreover, some studies have examined *state*‐level prejudices (i.e., prejudices that appear and disappear along with situations and circumstances) (Yilmaz et al., 2016) whereas our study examined *trait*‐level intolerance (dispositional intolerance, i.e., a personality trait that is comparatively stable over years). Hence, it may be that spiritual thinking may predispose to prejudice‐related responses in some situations (e.g., when primed by a certain religious stimulus; or in a certain situation where a target person has a religious orientation that is very uncommon in one's country) but not to a stable disposition to prejudices. Finally, the correlates of spiritual thinking may be partly culturally specific. In Finland, spiritual thinking is normatively at a comparatively low level, high scores correlating with adverse health outcomes such as higher likelihood of paranoid ideation (Saarinen et al., 2018). In the United States, for example, spiritual thinking is at a higher level than in some European countries (Farmer et al., 2003).

In this study, we found that participants’ or their parents’ education was not related to social intolerance. This was contrary to previous studies suggesting that low educational level is associated with lower antiracist attitudes, higher need for ethnic distance, and higher ethnocentrism (Meeusen & Dhont, 2015; Deary et al., 2008; Schoon et al., 2010; Hello et al., 2006). On one hand, our null results may be explained by that previous studies have focused on narrowly defined prejudices whereas we investigated social intolerance at a general level. On the other hand, there is a 9‐year compulsory comprehensive school in Finland that may result in less variance in educational level (when compared to other countries with different educational systems). In this study, we obtained no differences in social in tolerance between participants with comprehensive school, high school or occupational school, or academic level as highest completed education. Deary et al.’s (2008), for example, measured also lower classes of educational level (e.g., “no qualifications”) and found a link between low education and prejudices.

This study had some limitations. First, our data set does not allow making any conclusions about causal relationships of social intolerance with cognitive performance, socioeconomic factors, or cognitive styles. For example, it may be that very strong social intolerance may modify one's cognitive styles: stereotype threat (i.e., a threat of confirming negative in‐group stereotypes) is found to predict higher inflexible perseverance in cognitive tasks (Carr & Steele, 2009). Second, although cognitive performance was measured with the CANTAB that is a standardized and widely used international test battery, our test battery did not evaluate verbal performance. A meta‐analysis, nevertheless, indicated that high writing or reading abilities are not related to prejudice (Onraet et al., 2015). Nevertheless, many other studies on the topic have assessed full intelligence quotient (IQ) or more stable cognitive abilities (not merely cognitive performance during a single test situation). Third, we obtained a modest attrition bias so that included participants had slightly higher cognitive performance than dropped‐out participants (i.e., participants who had not data on, for example, social intolerance and could not be included in the analyses). Finally, our results cannot be directly generalized to different populations. As our sample consisted of ethnic Finns (not other ethnicities or ethnic minority members), our results may not be generalized to populations with different ethnicities. Circa 8.0% of the Finnish population are immigrants (Finland), while the corresponding percentage in the United States is 13.6% (OECD, 2020). Although our study did not focus on intolerance toward ethnic or racial outgroups, it is possible that contacts with different ethnicities in every‐day life might increase also broader intolerance toward different social groups.

Regarding practical implications, our study suggests that merely raising educational level or providing cognitive training may not be the most effective ways to reduce social intolerance in Western countries such as Finland. To date, a large proportion of prejudice‐related interventions appear to have directly targeted prejudice‐related contents (e.g., to provide knowledge about different minorities) (Rutland & Killen, 2015) but such prejudice‐directed interventions may even increase prejudices in some groups of individuals (Vorauer & Sasaki, 2010). Our findings tentatively suggest that targeting certain cognitive styles (i.e., how an individual processes acquired knowledge) would more effectively diminish social intolerance, if supposing that there might be some causal relationships between cognitive styles and social intolerance. Hence, it would be effective to focus on cognitive styles (without necessarily a direct link to contents of prejudices) in prejudice‐reduction interventions. In particular, when tailoring prejudice‐reduction interventions, it is important to consider that reducing perseverance and increasing flexibility and persistence of thinking could alleviate social intolerance. This is in accordance with a previous intervention study showing that increasing flexibility can effectively alleviate prejudices (Masuda et al., 2012).

There is promising evidence that cognitive styles can be enhanced in comparatively short‐term and cost‐effective interventions: for example, flexibility can be enhanced in 2–3 months (Fledderus et al., 2010; Puolakanaho et al., 2020) or even with a 1.5‐h‐long computer‐based program (Waller et al., 2011). This evidence comes, however, mostly from psychiatric populations, such as patients experiencing delusions, psychological distress, or burnout (Fledderus et al., 2010; Puolakanaho et al., 2020; Waller et al., 2011). Future studies could investigate whether these kinds of programs could be applied to nonclinical populations in interventions reducing social intolerance.

## FUNDING

The Young Finns Study has been financially supported by the Academy of Finland: grants 322098, 286284, 134309 (Eye), 126925, 121584, 124282, 129378 (Salve), 117787 (Gendi), and 41071 (Skidi); the Social Insurance Institution of Finland; Competitive State Research Financing of the Expert Responsibility area of Kuopio, Tampere and Turku University Hospitals (grant X51001); Juho Vainio Foundation; Paavo Nurmi Foundation; Finnish Foundation for Cardiovascular Research; Finnish Cultural Foundation; The Sigrid Juselius Foundation; Tampere Tuberculosis Foundation; Emil Aaltonen Foundation; Yrjö Jahnsson Foundation; Signe and Ane Gyllenberg Foundation; Diabetes Research Foundation of Finnish Diabetes Association. This project has received funding from the European Union's Horizon 2020 research and innovation programme under grant agreements No 848146 for To Aition and grant agreement 755320 for TAXINOMISIS; European Research Council (grant 742927 for MULTIEPIGEN project); Tampere University Hospital Supporting Foundation and Finnish Society of Clinical Chemistry.

### PEER REVIEW

The peer review history for this article is available at https://publons.com/publon/10.1002/brb3.2704

## Supporting information

Supplementary Information

## Data Availability

The Cardiovascular Risk in Young Finns (YFS) data set comprises health‐related participant data and their use is therefore restricted under the regulations on professional secrecy (Act on the Openness of Government Activities, 612/1999) and on sensitive personal data (Personal Data Act, 523/1999, implementing the EU data protection directive 95/46/EC). Due to these legal restrictions, the data from this study cannot be stored in public repositories or otherwise made publicly available. However, data access may be permitted on a case by case basis upon request. Data sharing outside the group is done in collaboration with YFS group and requires a data‐sharing agreement. Investigators can submit an expression of interest to the chairman of the publication committee (Prof. Mika Kähönen, Tampere University, Finland). The data sets presented in this article are not readily available because YFS is an ongoing follow‐up study and the data sets are not anonymized, and the GDPR prevents public sharing of the data. Instead, pseudonymized data sets are possible to share on request, and requires a data sharing agreement between the parties. Requests to access the data sets should be directed to Liisa Keltikangas‐Järvinen (liisa.keltikangas-jarvinen@helsinki.fi) for the data on social intolerance, to Katri Räikkönen (katri.raikkonen@helsinki.fi) or Niklas Ravaja (niklas.ravaja@helsinki.fi) for other psychological data, to Terho Lehtimäki (terho.lehtimaki@tuni.fi) for genetic data set, and to Suvi Rovio (suvrov@utu.fi) for CANTAB data set.
