# Supplementary material for: Does social intolerance vary according to cognitive styles, genetic cognitive capacity, or education?
Source: Brain Behav. 2022 Sep 1;12(9):e2704. doi: 10.1002/brb3.2704 (PMC9480910; doi:10.1002/brb3.2704)
Supplement: Supplementary file 1 — Supplementary Information [file BRB3-12-e2704-s001.docx]

**Supplementary Material. The results of attrition analyses.**

Included participants were slightly older (27.6 vs. 27.3, p<.05) and more likely men (57.9% vs. 43.0%, p<.001) than excluded participants. Included participants had slightly higher flexibility (3.02 vs. 2.95, p<.05), higher polygenic cognitive potential (0.03 vs. -0.07, p<.05), and higher performance in the Paired Associates Learning Test (0.03 vs. -0.12, p<.01) and in the Rapid Visual Processing Test (0.05 vs. -0.17, p<.001). Included participants’ had slightly higher home income in childhood (4.88 vs. 4.69, p<.01) and more likely high educational level (38.8% vs. 12.9%, p<.001) than excluded participants. There was no attrition bias in social intolerance, flexibility, perseverance, persistence, non-rational thinking, performance in the Reaction Time Test, performance in the Spatial Working Memory Test, participants’ level of income in adulthood, or parents’ educational level.

**Supplementary Table 1.** The results of the growth curve model when predicting the trajectory of social intolerance by all the predictors simultaneously.

|  | B | SE | *p* |
| --- | --- | --- | --- |
| Distractibility | 0.006 | 0.036 | 0.862 |
| Persistence | -0.139 | 0.041 | **0.001** |
| Perseverance | 0.482 | 0.140 | **0.001** |
| Flexibility | -0.629 | 0.042 | **< 0.001** |
| Non-rational thinking | -0.023 | 0.032 | 0.469 |
| Paired Associates Learning Test | 0.023 | 0.026 | 0.378 |
| Spatial Working Memory Test | 0.027 | 0.026 | 0.961 |
| Rapid Visual Information Processing Test | 0.016 | 0.027 | 0.378 |
| Reaction Time Test | -0.001 | 0.025 | 0.961 |
| Polygenic cognitive potential | 0.034 | 0.024 | 0.159 |
| Family income in childhood | 0.005 | 0.015 | 0.711 |
| Parents’ educational level | 0.046 | 0.038 | 0.226 |
| Participants’ level of income | -0.004 | 0.009 | 0.647 |
| Participants’ educational level | 0.050 | 0.050 | 0.317 |
| Note: Adjusted for participants’ age and sex. B coefficients with standard errors (SEs) and *p* values refer to the estimated fixed effects.  *n* = 922 | | | |
